# Introduction to the 2019 World Federation for Medical Education World Conference

**DOI:** 10.3352/jeehp.2019.16.1

**Published:** 2019-01-02

**Authors:** Duck-Sun Ahn

**Affiliations:** 1 Vice-President, World Federation for Medical Education, Ferney-Voltaire, France; 2 Chair, Organizing Committee of the 2019 World Federation for Medical Education World Conference, Seoul, Korea; Hallym University, Korea

Date: April 7–11, 2019

Venue: Grand Walkerhill Seoul, 177 Walkerhill-ro, Gwangjin-gu, Seoul 04963, Korea

Homepage: http://wfme2019.org

Online Registration Due: March 29, 2019

## World Federation for Medical Education

The World Federation for Medical Education (WFME) was established in 1972 by the World Medical Association (WMA) and the World Health Organization (WHO). It is a worldwide organization dedicated to improving the quality of medical education. It also has 6 regional associations of medical education, which correspond to the same areas as the 6 regional organizations of the WHO. Korea is affiliated with the Western Pacific Association for Medical Education, the association of the Western Pacific region, as part of the WHO’s Western Pacific region. The current president of the WFME is Dr. David Gordon, a graduate of the University of Cambridge, United Kingdom, who has served as president of the University of Manchester School of Medicine, president of the Medical School Council, and president of the Association of Medical Schools in Europe.

The purpose of the WFME is to provide an international forum for improving the quality of medical education. Its major achievements were the ‘Edinburgh Declaration’ in 1988, which became a watershed of medical education reforms internationally, followed by the ‘World Summit and Recommendation in Edinburgh’ in 1993. Subsequently, reforms in medical education based on the ‘Recommendation’ have been widespread throughout the world.

The 2003 World Conference of the WFME in Copenhagen was particularly significant. Medical educators from all over the world attended this conference, after which the WFME published accreditation criteria and standards for basic medical education, postgraduate medical education, and continuing professional development. Since then, it has been adopted as the standard for the evaluation of medical colleges around the world. In addition, the accreditation criteria for postgraduate medical education have been adopted by the World Organization of National Colleges, Academies and Academic Associations of General Practitioners/Family Physicians (WONCA) as a basis for their course programs. The WONCA has already launched an international accreditation program for training courses in family medicine. In 2015, the WFME moved from its Copenhagen office to its current location in Ferney-Voltaire near Geneva.

Currently, the WFME Executive Committee consists of the former president, president, vice-president, presidents of 6 regional organizations, and representatives from the WHO, WMA, Educational Commission for Foreign Medical Graduates, International Federation of Medical Students’ Associations, and Junior Doctor Network, which function as medical education associations for all physicians throughout the world.

## Three core activities of the World Federation for Medical Education

First, it recognizes institutions in each country that work for the accreditation of medical schools.

Second, it pursues initiatives to enhance all 3 stages of medical education: basic medical education, postgraduate medical education, and continuing professional development.

Third, it publishes the World Directory of Medical Schools and maintains its contents.

The WFME is playing an increasingly important role in providing institutional recognition for medical education accreditation institutions in each country. For example, the Korea Institute of Medical Education and Evaluation was the fourth-recognized national institution in the world in 2015 by WFME. It has been designated as the official medical evaluation system of Korea by the Ministry of Education of the Republic of Korea.

## World Federation for Medical Education Conference

### 2003 World Conference

The WFME has traditionally held academic conferences on an irregular basis, approximately every 10 years. The most recent one was held in Copenhagen, Denmark in 2003. Lund University, the University of Copenhagen in Denmark, and the governments of Sweden and Denmark provided budgetary support to the Secretariat of the WFME. In addition, the United Nations, the WHO, the UNESCO (United Nations Educational, Scientific and Cultural Organization), and the WMA cooperated with each other to ensure that the WFME Conference was successfully held in 2003. More than 700 medical education experts from 75 countries participated in the 2003 conference. Approximately 30 professors from medical schools in Korea participated. After the conference, they established the basis of the accreditation system of medical schools in Korea, which has been implemented since then.

### 2013 World Conference

The 2013 World Conference was planned to be held in Malmö, Sweden in 2013; however, due to the economic crisis in Europe that started in Greece in 2013, it was canceled.

### 2019 World Conference

The WFME has recently been moving away from the United States and Europe-centered organizations to promote medical education development from a broader and more global perspective. The WFME has expressed interest in Korea’s economic growth and the development of medicine in Korea; furthermore, it confirmed the plan to host the next academic conference in Korea at the WFME Implementation Conference held in May 2017.

## Anticipated effects

The WFME Conference has never been held outside Europe and the United States. In this context, holding the WFME Conference in Korea is expected to open a new horizon for medical education in Korea and Asia in the 21st century. I am confident that it will be a good opportunity to promote Korea’s international status, as well as to raise awareness of the leading role that East Asia plays in medical education. In particular, the conference is expected to include an event that will provide a breakthrough opportunity for improving the education of residents in Asia. In addition, I believe that it will serve as a very useful opportunity to improve the level of medical education in many countries through its coverage of various content areas, such as medical education evaluation accreditation, licensing examinations, and continuing education.

